# Assessing the oral and suprahyoid muscles in healthy adults using muscle ultrasound to inform the swallowing process: a proof-of-concept study

**DOI:** 10.1038/s41598-024-62032-z

**Published:** 2024-06-08

**Authors:** Eileen Kelly, Saira Nazeer, Brigitta Fazzini, Anna-Liisa Sutt, Segun Olusanya, Thomas Campion, Zudin Puthucheary

**Affiliations:** 1 Adult Critical Care Unit, The Royal London Hospital, Barts Health NHS Trust, London, E1 1BB UK; 2 Critical Care and Perioperative Medicine Research Group, William Harvey Research Institute, Queen Mary University of London, London, UK; 3 Department of Speech and Language Therapy, The Royal London Hospital, Barts Health NHS Trust, London, UK; 4 Critical Care Research Group, Institute of Molecular Bioscience, University of Queensland, Brisbane, Australia; 5 Consultant in Intensive Care Medicine and ECMO, Department of Perioperative Medicine, Barts Heart Centre, London, UK; 6 Consultant Head and Neck Neck/Neuroradiologist, The Royal London Hospital, Barts Health NHS Trust, London, UK

**Keywords:** Oral suprahyoid muscles, Ultrasound, Muscle mass, Muscle function, Medical research, Diagnosis

## Abstract

The oral and suprahyoid muscles are responsible for movements of swallowing. Our study aimed to determine the reproducibility of static and dynamic measurements of these muscles using bedside ultrasound equipment. Forty healthy participants were recruited prospectively. Primary outcomes were evaluation of mass measurements of the anterior bellies of the digastric, mylohyoid, geniohyoid and tongue in B-mode ultrasound. Secondary outcomes were evaluation of geniohyoid muscle layer thickness and function using M-mode. Muscle mass measurements demonstrated little within-participant variability. Coefficient of Variance (CoV) across muscles were: anterior belly digastric (5.0%), mylohyoid (8.7%), geniohyoid (5.0%) and tongue (3.2%). A relationship between sex (r^2^ = 0.131 *p* = 0.022) was demonstrated for the geniohyoid muscle, with males having higher transverse Cross Sectional Area (CSA) (14.3 ± 3.6 mm vs. 11.9 ± 2.5 mm, *p* = 0.002). Tongue size was correlated with weight (r^2^ = 0.356, *p* = 0.001), height (r^2^ = 0.156, *p* = 0.012) and sex (r^2^ = 0.196, *p* = 0.004). Resting thickness of the geniohyoid muscle layer changed with increasing bolus sizes (f = 3.898, *p* = 0.026). Velocity increased with bolus size (*p* =  < 0.001, F = 8.974). However swallow time and slope distance did not, potentially influenced by higher coefficients of variation. Oral and suprahyoid muscle mass are easily assessed using bedside ultrasound. Ultrasound may provide new information about muscle mass and function during swallowing.

## Introduction

Dysphagia is a dysfunction of one or more parts of the swallow and can occur due to physiological or structural abnormalities^1^. Dysphagia is commonly reported in acutely unwell patients, with incidence rates ranging from 12 to 80%^2–4^. Clinical complications of dysphagia include mortality, aspiration pneumonia, dependence for tube feeding, increased hospital length of stay and cost^2,5–7^. Dysphagia also negatively affects patients quality of life and psychosocial wellbeing^8^.

A safe and efficient swallow requires coordination of the oropharyngeal muscles, cranial nerves, cerebral cortex, medulla oblongata and the muscles of respiration^9,10^. Swallowing is a synergistic process, requiring the precise timing of biomechanical movements as food or fluid transfer from the oral cavity through the pharynx to the oesophagus^11,12^. The sequence of normal swallowing is preceded by contraction of the oral and suprahyoid musculature, followed by the contraction of the pharyngeal constrictor muscles and the relaxation of the cricopharyngeal muscles^13^. The oral and suprahyoid muscles are responsible for the initiation of the swallow and the upward movement of the hyoid bone, while the pharyngeal muscles are responsible for the movement of the bolus through the oesophagus^14^. Impairment can arise from dyscoordination of movements, resulting in a diminished ability to safely and efficiently transfer the bolus from the oral cavity to the oesophagus^11^.

Loss of muscle mass is known to result in functional impairment^15,16^ and has been shown to negatively affect swallow function in different disease states, such as Motor Neurone Disease, Parkinson’s Disease and Myotonic Dystrophy^17–19^. Ultrasound has been used to assess morphometry of muscle groups related to swallowing^20^. Evaluation includes measurements of muscle thickness, cross-sectional area, contraction and gradings of muscle echogenicity^18^. Examining changes to the structure and quality of oral and suprahyoid muscles over time may enable a greater understanding of the mechanism of muscle wasting on swallow function. While the oral and suprahyoid muscles can be easily imaged using ultrasound, there are little data on the reliability and repeatability of these measures, the effect of training on reliability and repeatability and fewer data on the use of dynamic ultrasound to evaluate muscle function^21^. We set out to determine the reproducibility of static and dynamic measurements of the oral and suprahyoid muscles using bedside ultrasound equipment.

## Aim

The aim of this research was to establish the proof-of-concept of using muscle ultrasound to evaluate the oral and suprahyoid muscles in healthy participants. The primary outcomes were to assess muscle mass measurements of the anterior bellies of the digastric muscles, mylohyoid muscle, geniohyoid muscle and tongue, determine the coefficient of variation between muscles and evaluate relationships between participant age, sex height and weight. The secondary outcomes was to evaluate functional measures of the geniohyoid muscle during swallowing.

## Methods

### Ethical approval

The study was approved by Queen Mary University of London ethics committee (reference number: QMERC23.063). All methods were performed in accordance with relevant guidelines and regulations and in accordance with the Declaration of Helsinki.

### Participants

Participants were recruited via email advertisement with accompanying participant information sheet. Adults > 18 years without pre-existing surgery to the head or neck or known laryngeal pathology (e.g., vocal fold palsy) and with self-reported normal swallowing function were included. Informed consent was obtained prior to participation. Participants provided baseline demographics (e.g., age, sex and height).

### Study design and protocol development

A prospective observational study was designed. Protocol was developed prior to the study by a Speech and Language Therapist (EK), consultant Head and Neck Radiologist (TC) and muscle physiologists (ZP & SO). Measurements of muscle mass were selected based on previous research^22^ and included cross-sectional area (CSA), height and width. Parameters to evaluate muscle function were based on previous research^18,23,24^ and involved examining geniohyoid muscle layer thickness (at rest and maximum contraction), velocity, slope distance and swallow time. We selected four boluses (10, 20, 30 and 40 ml) to examine for responsiveness to increasing volume. The geniohyoid was selected as the muscle of interest due to its role in the upward, anterior displacement of the hyoid and its ease of imaging. No formal competencies for ultrasound accreditation exist in the United Kingdom for Speech and Language Therapists, therefore the principal investigator (EK) was trained in ultrasound by a Consultant Head and Neck Radiologist (TC) and muscle physiologist (ZP) prior to commencement of the study. This included observation, practical supervision and independent examination over 12 months acquiring over 50 scans to gain independent proficiency level.

### Ultrasound equipment

A GE Venue™ ultrasound machine was used for all image acquisition and analysis. Analysis of muscle mass and muscle function took place on the GE Venue™ ultrasound machine using its internal measurement software. This was carried out by the principal investigator. Depth and gain were pre-set prior to each examination. Images were anonymised and stored on the ultrasound machine. Linear and curvilinear probes were used to obtain views in transverse and sagittal planes.

### Procedure

All examinations were carried out by the principal investigator (EK). Participants were seated in an upright position with head in neutral. The investigator was seated facing the ultrasound machine screen, with the ultrasound probe held in the right hand. Lubricant gel was generously applied to the probe to avoid excess pressure being applied on the participant’s skin. For the bolus trials, water (10, 20, 30 and 40 ml) was measured with a syringe and placed into labelled cups prior to the examination. Three images were taken for each parameter for each participant.

### Muscle mass

**Figure 1:**
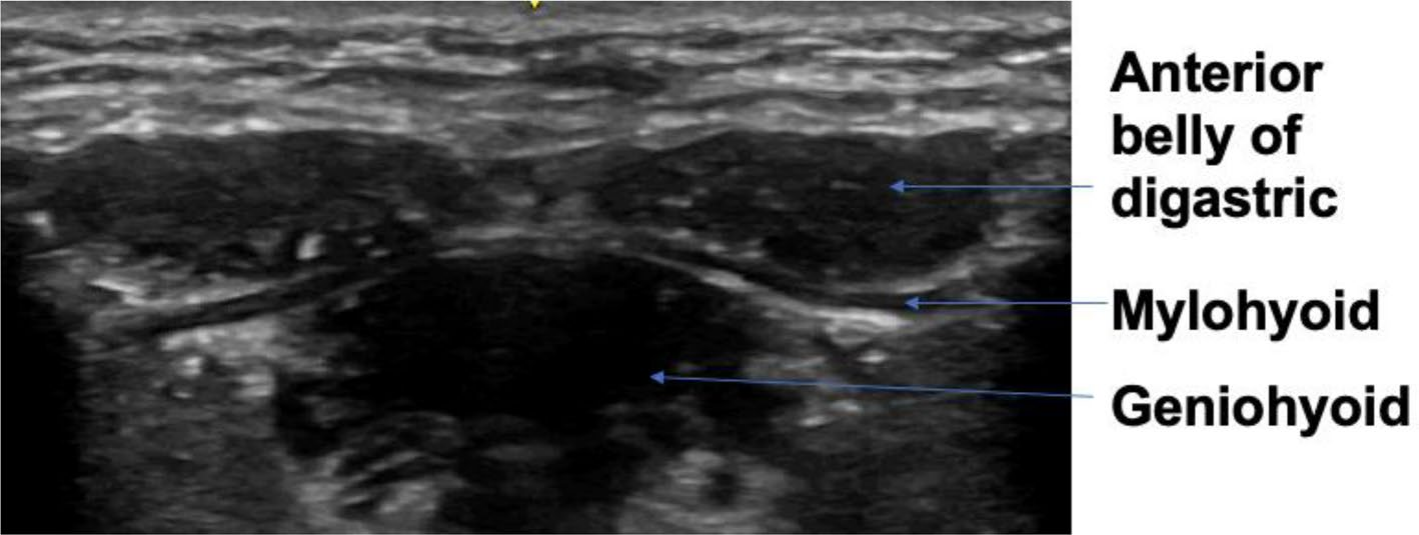
Mass measurements suprahyoid muscles.

**Figure 2:**
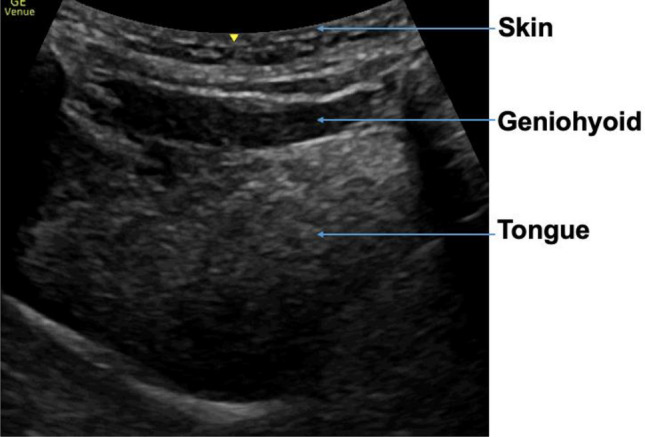
Mass measurements geniohyoid and tongue.

The muscles of interest for evaluation of muscle mass were the anterior bellies of the digastric muscles (left and right), mylohyoid muscle, geniohyoid muscle and the tongue. The linear probe was used for all muscle mass measurements in transverse plane, except for tongue CSA which was taken by the curvilinear probe in sagittal plane. The probe was placed in the midline transverse plane, 2 cm from the mandible symphysis. The anterior belly of the digastric muscle image was taken when both bellies were seen in their entirety. The mylohyoid was measured under the digastric muscle from the upper to lower boundary of the fascia. The geniohyoid was located immediately superior to the mylohyoid muscle. The CSA of the geniohyoid was also taken in sagittal plane, visible as a hypoechoic band between the mandible and hyoid bone. Tongue CSA was also taken in the sagittal plane. The tongue is bordered superiorly by a hyperechoic dorsal surface, inferiorly by the fascia, and anterior-posteriorly by the mandible and hyoid bone. Figures 1 and 2 illustrates the muscle mass image taken.

### Muscle function

**Figure 3:**
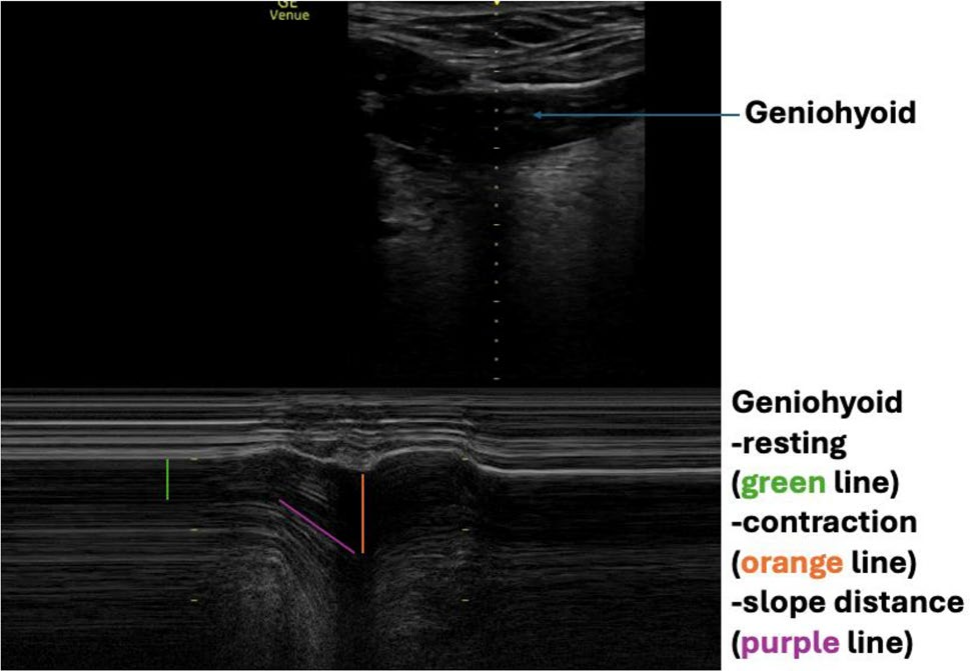
Geniohyoid muscle function (resting and contraction).

The geniohyoid was selected for analysis of muscle function. The geniohyoid is bordered by the mandible and hyoid bone anteriorly and posteriorly, and by the mylohyoid and tongue superiorly and inferiorly. The M-mode line was placed in the midline of the geniohyoid muscle. All participants took three boluses of water, across each bolus size (10–40 ml). Participants were instructed to hold the bolus in their mouth until prompted to swallow the bolus in one swallow. Offline analysis of resting and maximum contraction of the geniohyoid muscle layer thickness took place after the assessment. Resting geniohyoid muscle and maximum contraction layer thickness were measured using the measurement tool (calliper function). Additional measures of velocity, swallow time and slope distance were obtained. Velocity was measured by calculating the slope distance divided by the slope time. Swallow time was measured by calculating the time taken for the geniohyoid muscle layer to depress from resting position to maximum contraction and return to baseline. Slope distance was measured by calculating the distance between the edge of the angle of the geniohyoid muscle layer at resting position and the deepest point of the geniohyoid muscle layer thickness during maximum contraction. A step-by-step measurement process is outlined in Supplementary Material S1. Figure 3 illustrates the muscle function image taken.

### Data collection

Measurements were recorded in an anonymised Microsoft Excel™ spreadsheet. Values were recorded for each participant, with each participant having three values for each measure (e.g., CSA, velocity, swallow time). The average value of three consecutive images was used in analysis. The CSA, height and width were recorded for each muscle (mm^2^ and mm). Measurements of geniohyoid muscle layer thickness were taken at rest and at maximum contraction. Muscle layer thickness (mm), slope distance (mm), slope time (seconds), velocity (mm/s) and swallow time (seconds) were recorded for each bolus. When parameters were missing data, this was recorded as such on the spreadsheet.

### Statistical analysis

Data for demographic information, muscle mass CSA and geniohyoid muscle layer thickness were assessed using descriptive statistics (e.g., mean, standard deviation (SD)). Muscle mass data were examined for relationships between demographic variables (e.g., sex, height) using univariate analysis. Coefficient of variation (CoV) was calculated for muscle mass and geniohyoid layer thickness to measure variability. Correlation coefficient was used to denote the association between muscle CSA. To assess the impact of increasing bolus sizes on geniohyoid muscle layer thickness, one-way repeated measures ANOVA was carried out. Data were tested for normality using the D’Augustino and Pearson normality test.

## Results

### Participant characteristics

A convenience sample of forty healthy participants were recruited for this study between October 2023 and January 2024. Demographic data (age, height, sex) was collected for all participants n = 40, except for weight which was collected from n = 28 participants. Summary Table 1 represents mean values for the included participants.

**Table 1:** Participant demographic information. Data are mean (range).

Sex	Female n = 29	Male n = 11
Age (years)	35 (26–56)	35 (27–46)
Height (cm)	168 (157–180)	181 (172–191)
Weight(kg)*	63 (49–82)	80 (68–90)

N = 40 except for when indicated by *(n = 23 females, n = 5 males).

### Oral and suprahyoid muscle mass measurements

#### Anterior belly of the digastric

The mean (± SD) CSA of the left and right anterior belly of the digastric were highly correlated (r = 1.0, *p* < 0.001) and were 7.0 ± 1.7mm^2^ and 7.0 ± 1.7mm^2^ respectively. No relationship was seen between age, height, weight, sex and CSA in univariate analyses (r^2^ < 0.1 for all).

#### Mylohyoid

The mean (± SD) CSA was 5.9 ± 2.0 mm (n = 30). No relationship was seen between age, height, weight, sex and CSA in univariate analyses (r^2^ < 0.1 for all).

#### Geniohyoid

**Figure 4:**
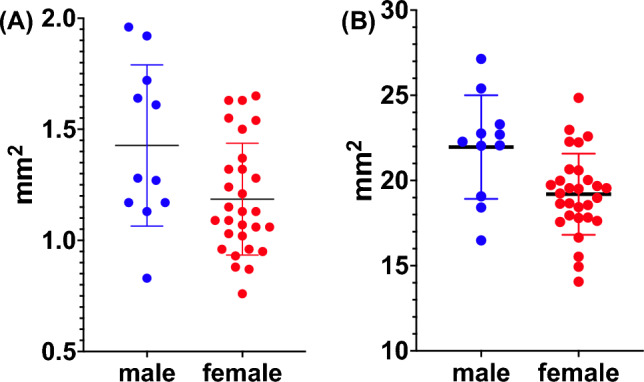
Geniohyoid (**A**) and Tongue (**B**) Cross-Sectional Area between sexes.

The mean (± SD) CSA of the geniohyoid in transverse and sagittal planes was correlated (r = 0.398, *p* = 0.030), 12.5 ± 3.0 mm (n = 40) and 27.5 ± 4.7 mm (n = 30). No relationship was seen between age, height, weight and transverse CSA in univariate analyses of (r^2^ < 0.1 for all). A relationship was seen with sex (r^2^ = 0.131 *p* = 0.022) and males had a higher transverse CSA than females (14.3 ± 3.6 mm vs. 11.9 ± 2.5 mm, *p* = 0.002, Fig. 4a). Geniohyoid and mylohyoid mass measurements correlated (r = 0.443, *p* < 0.05).

#### Tongue

The mean (± SD) CSA was 199.6 ± 28.4 mm. Tongue size was correlated with weight (r^2^ = 0.356, *p* = 0.001), height (r^2^ = 0.156, *p* = 0.012) and sex (r^2^ = 0.196, *p* = 0.004, Fig. 4b) but not age (r^2^ < 0.1).

Correlation between muscle mass measurements across the assessed muscles is included in Table 2.

**Table 2:** Correlation between different muscle masses (*p < 0.05).

	Anterior belly digastric left	Mylohyoid	Geniohyoid	Tongue
Anterior belly digastric left	–	.116	0.170	0.065
Mylohyoid	0.116	–	0.443*	− 0.211
Geniohyoid	0.170	0.443*	–	0.062
Tongue	0.065	− 0.211	0.062	–

Significant values are in bold.

### Functional measurements of geniohyoid muscle

#### Geniohyoid muscle layer thickness: resting versus contraction

Resting thickness of the geniohyoid muscle layer changed with increasing bolus sizes (10–40 ml) (f = 3.898, *p* = 0.026). A difference between resting (7.2 ± 1.7 mm) and contraction thickness (11.8 ± 2.6 mm) was noted (4.54 ± 2.0 mm *p* =  < 0.001). However, no relationship was found between bolus size and change in thickness (*p* = 0.191).

#### M-mode measurements of swallow function

##### Swallow time

Mean swallow time was 1.44 ± 0.3 s (n = 30). The mean time per group (10–40 ml) was 1.4 ± 0.18 s, 1.44 ± 0.26 s, 1.41 ± 0.27 s and 1.50 ± 0.49 s respectively. Swallow time did not alter between boluses (*p* = 0.307, F = 1.176).

##### Velocity

Mean velocity was 22.8 ± 7.3 mm/s (n = 40). The mean velocity increased with bolus size (*p* =  < 0.001, F = 8.974) and were 18.15. ± 3 mm/s (10 ml), 22.5 ± 6.7 mm/s (20 ml), 24.1 ± 7.3 mm/s, (30 ml) and 26.0 ± 7.2 mm (40 ml). Neither tongue CSA nor geniohyoid CSA were predictors of velocity (*p* = 0.922 and *p* = 0.345).

##### Slope distance

Mean slope distance was 19.9 ± 4.4 mm (n = 35). Slope distance did not differ between groups (*p* = 0.948, F = 0.120). Geniohyoid CSA was a predictor of slope distance (F = 15.302, *p* =  < 0.001). Tongue CSA was not a predictor of slope distance (F = 0.671, *p* = 0.414).

##### Training effect

**Figure 5:**
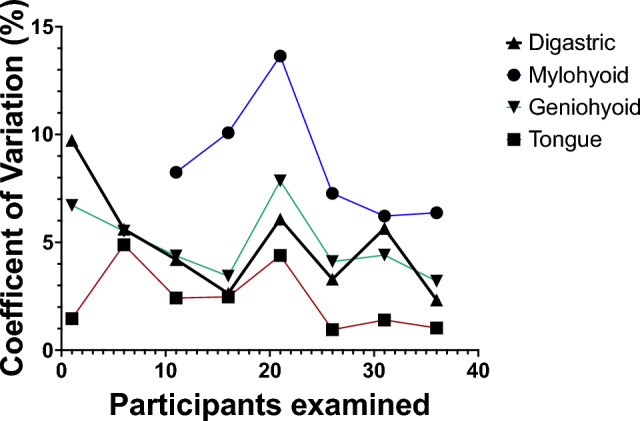
Coefficient of Variation across participants for different muscle groups.

The CoV was calculated for muscle mass and geniohyoid muscle layer thickness. The CoV for all muscles CSA was below 5%, except for the mylohyoid CSA which was 8.6%. All CoV were calculated based on n = 40 participants, except for mylohyoid (n = 30). To determine if a training effect took place, CoV was calculated at intervals (e.g., participants 1–5, 5–10). Table 3 illustrates decreasing CoV over time in all muscles. Figure 5 illustrates CoV across participants for different muscle groups. CoV was also calculated for assessments of muscle function. CoV for resting muscle layer thickness ranged from 11.15 to 13.6%, contracting 9.81–12.33%, velocity 21.5–23.6%, slope distance 6.56–9.80% and swallow time 12.1–16.3%. CoV did not decrease over time when participants were analysed by group.

**Table 3:** Coefficient of variation of muscle CSA.

	ABDleft	Mylohyoid	Geniohyoid	Tongue
CoV across all participants				
1–40*	4.9	8.6*	4.9	3.2
Breakdown per group				
1–5	9.7		6.7	1.5
6–10	5.6		5.5	4.9
11–15	4.2	8.3	4.4	2.4
16–20	2.6	10.1	3.4	2.5
21–25	6.1	13.6	7.9	4.4
26–30	3.2	7.3	4.1	1.0
31–35	5.7	6.2	4.4	1.4
36–40	2.3	6.4	3.2	1.0

n = 40 participants. *Denotes n = 30 participants.

## Discussion

This proof-of-concept study evaluated muscle mass of the oral and suprahyoid muscles and geniohyoid muscle function. We found a relationship between participant weight, height, sex and the CSA of the tongue. Participant sex was associated with geniohyoid CSA. CSA was not closely correlated across muscle groups examined. Oral and suprahyoid muscles were easily scanned, with little variance between measurements of mass and an observable training effect. Muscle layer thickness altered between resting and maximum contraction during bolus trials, however this measure was not responsive to bolus size. In regard to dynamic measures, only velocity altered significantly across bolus sizes, though there was variability in the CoV across all dynamic measures.

### Implications of muscle mass measurements

We demonstrated that the oral and suprahyoid muscles are easily visualised with bedside ultrasound equipment. We found a correlation between sex, weight and tongue CSA, which is consistent with previous research^25,26^. Previous research has identified the association between muscle wasting and dysphagia, namely prolonged oral preparatory and transit time^27^, impaired swallow safety resulting in penetration/aspiration^28^ and reduced hyolaryngeal approximation^29,30^. Muscle wasting occurs in critically unwell patients, with muscles such as the rectus femoris and biceps branchii shown to waste at a rate of 2% per day during acute illness^31^. Although the fibre compositions of the oral and suprahyoid muscles are different from those of limb muscles^32^, the oral and suprahyoid muscles consist predominately of type II muscle fibres^32–34^ which are known to experience preferential loss during acute muscle wasting^31,35,36^. It is likely that given the variance in mass, oral and suprahyoid muscles waste at different rates. While the muscles of swallowing work as a functional unit, each muscle plays an individual and differential role in swallowing^37^, and wasting at different rates may result in dyscoordination of the swallow. The intrinsic and extrinsic muscles of the tongue contract and pull it forward (genioglossus), backward and downwards (hyoglossus) and coordinate the fine movements involved in changing tongue shape^38^. The digastric muscles aid jaw movement and elevate the hyoid bone, while the mylohyoid and geniohyoid muscle pull the hyoid upwards and forwards^37–39^. Wasting to one or all of these muscles may result in impairment of the swallow. Identifying which muscles waste, and at what rate, will enhance specificity when diagnosing and treating swallow dysfunction.

### Implications of dynamic measurements

Changes to the geniohyoid muscle layer thickness from resting position to maximal contraction was reliably detected by ultrasound. The use of M mode to quantify muscle thickness has been used widely to demonstrate changes to muscle^15,31,40^. Dynamic measurements of swallowing on ultrasound have typically investigated hyoid movement during swallow tasks^30,41,42^. We sought to investigate the dynamic movements of the geniohyoid muscle, to understand if increasing bolus size could elicit changes to velocity, slope distance and swallow time. These parameters were selected to probe the geniohyoid muscle, which is easily imaged on ultrasound, and to determine if future work could examine the influence of strain on the muscle.

Velocity of the geniohyoid was influenced by the bolus size, with a two-fold increase detected as the bolus sizes progressed. This is consistent with previous research examining changes in hyoid bone velocity across bolus sizes^43^. There is potential that in patients with muscle wasting, the force and speed of muscle contraction may be altered and this may result in dyscoordination of the swallow. However, a key finding was the wide range of CoV with functional measures, in contrast to the measures of muscle mass. Further investigation is warranted to determine if this requires greater training of the assessor, as is reflected in other ultrasound frameworks^44^, or whether it is related to the volitional nature of prandial swallowing and within-participant variability. Surprisingly, our data did not detect variance in swallow times across bolus sizes. This may be due to the use of one bolus type (e.g., water only) or choosing a single parameter to evaluate this function (e.g., geniohyoid muscle layer). Future observational work may include both the time to maximum contraction and the displacement of the hyoid, to compare the ability of either parameter to detect a change in swallow time.

### Limitations

The primary limitation of this study is the imbalance in sex of the participants. Further, a smaller sample size limits the strength of correlations, and may be underpowered to detect changes. Our data included mostly younger females, a more variable participant sample would enable greater analysis of the relationship between demographic information and ultrasound measures.

### Future directions

Our data has shown static measures of the oral and suprahyoid muscles to be easily obtained with little within-participant variation. While our study only recruited healthy participants, there were demonstrated signals warranting future investigating patients with pathology. Examining static measures of muscle mass longitudinally may allow for a greater understanding of the relationship between mass, wasting and function. The oral and suprahyoid muscles are responsible for a range of core parameters leading to a safe and efficient swallow, such as manipulating the bolus, holding the bolus in the oral cavity and elevation of the hyoid bone. If muscle wasting occurs and at different rates, it may result in dyscoordination of the swallow. Determining an association between the mechanism of muscle wasting and underlying patterns of dysphagia will enhance evaluation and help tailor interventions.

Measurements of mass alone cannot convey changes to the function of the muscle. We sought to investigate functional parameters such as velocity and time, to understand patterns of dynamic movement within the muscles of interest. While our CoV was wide-ranging and requires further investigation before adapting to a clinical setting, we found a change in geniohyoid velocity across bolus sizes. Hyoid velocity has been shown to increase in individuals with reduced hyoid displacement and sarcopenia, potentially due to adaptation and compensation^45^. Investigating changes to velocity and/or additional measurements of function (e.g., strain, loading) will help us to understand what physiological changes have occurred to the muscle and how we can manipulate these in rehabilitation^46^. Ultrasound is uniquely primed to evaluate these parameters of muscle function, which are not captured in gold-standard instrumental evaluations such as videofluoroscopy or fibreoptic endoscopic evaluation of swallowing.

Finally, as the focus of dysphagia intervention has focused on neuro-modulation and skill-based therapy, we must investigate the role of muscle wasting on dyscoordination. If muscle wasting occurs across the functional unit, we must consider at what rate and to what extent. This will inform timing of interventions, which could lead to earlier intervention and/or the prevention of wasting.

## Conclusion

Oral and suprahyoid muscle mass are easily assessed using bedside ultrasound. Ultrasound may provide new information about muscle function during swallowing, however these findings require further research before being adopted into clinical practice.

### Supplementary Information

Supplementary Figures.

## Data Availability

Data to support the results reported in the article are available from the corresponding author on request.
