# Supplementary material for: Assessing the oral and suprahyoid muscles in healthy adults using muscle ultrasound to inform the swallowing process: a proof-of-concept study
Source: Sci Rep. 2024 Jun 8;14:13198. doi: 10.1038/s41598-024-62032-z (PMC11162466; doi:10.1038/s41598-024-62032-z)
Supplement: Supplementary file 1 — Supplementary Figures. [file 41598_2024_62032_MOESM1_ESM.docx]

**Supplementary Material S1.**

*Step-by-step measurement process*

All measurements were performed on a GE Venue^TM^ ultrasound machine. Measurements were calculated using the inbuilt measurement software.

1. **Resting geniohyoid thickness**

Resting geniohyoid thickness was measured by placing the calliper tool from top to bottom border of the fascia at the midline.

Figure A. Resting geniohyoid thickness


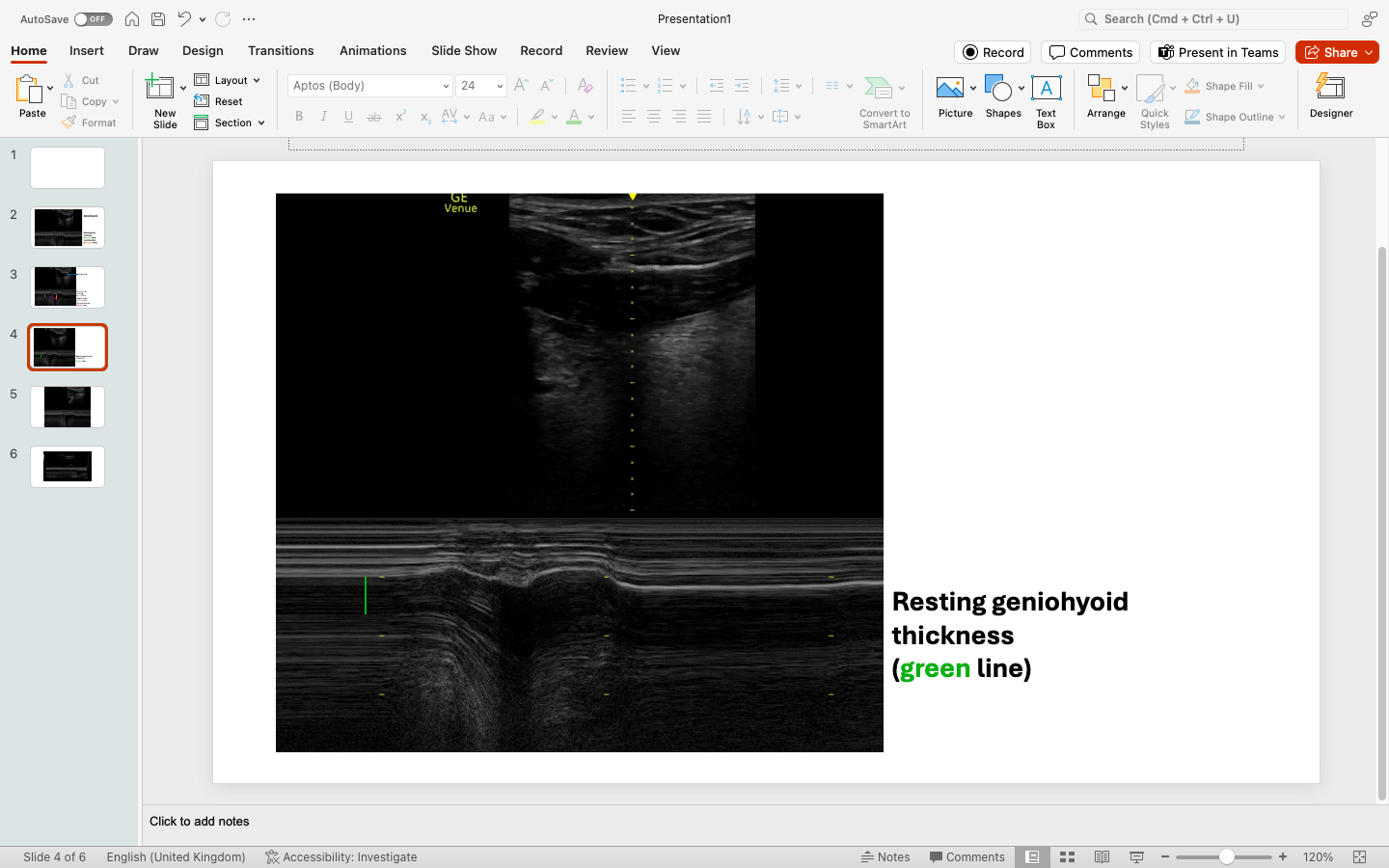


1. **Contraction thickness**


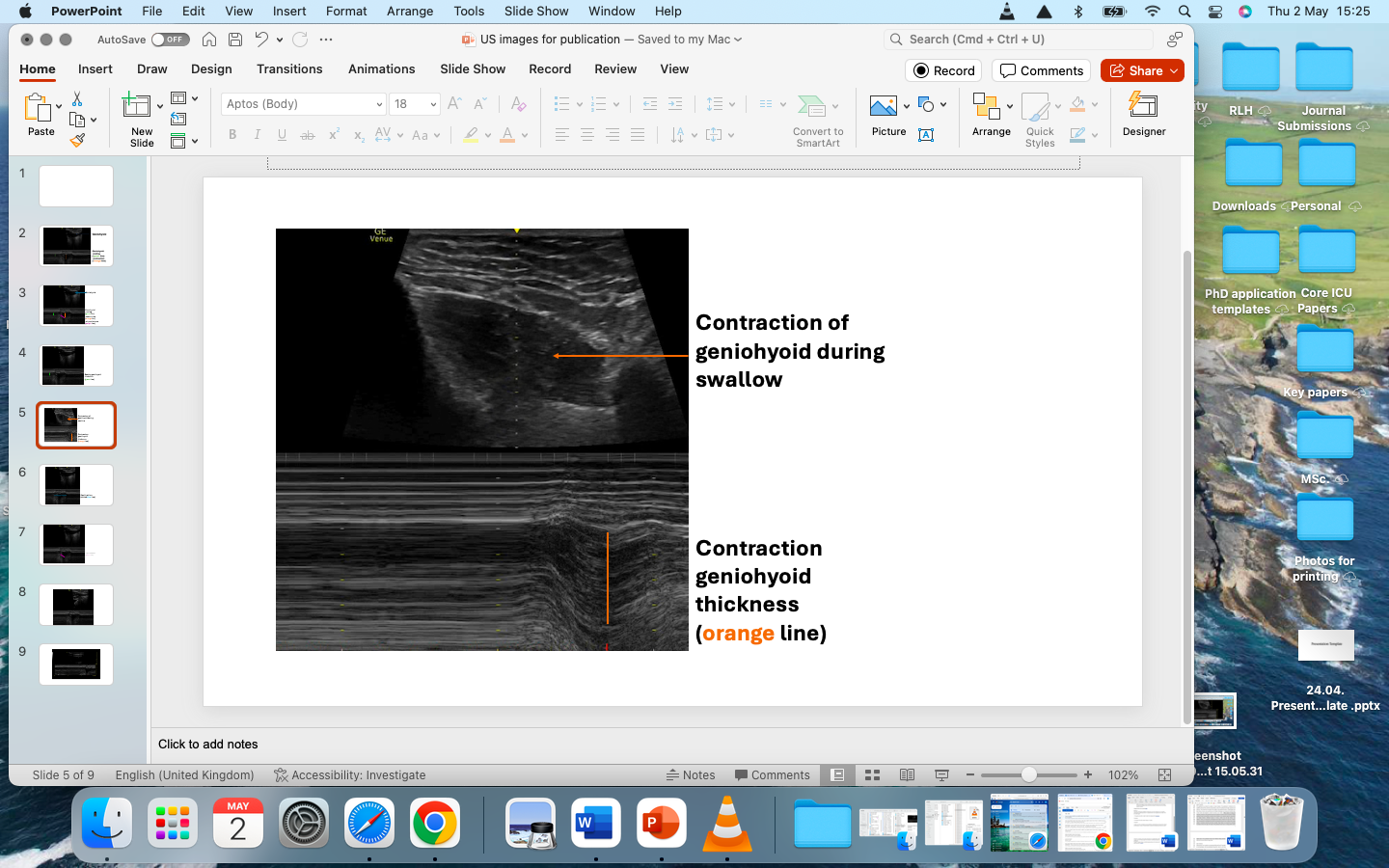
Contraction thickness was measured by placing the calliper tool from top to bottom border of the fascia during maximum contraction of the muscle. During the swallow, the geniohyoid contracts and shortens. Contraction thickness was measured at the deepest point of the muscle contraction.

Figure B. Contraction thickness

1. **Swallow time**

Swallow time was calculated by measuring the time taken for the geniohyoid muscle layer to depress from resting position to maximum contraction and return to baseline.


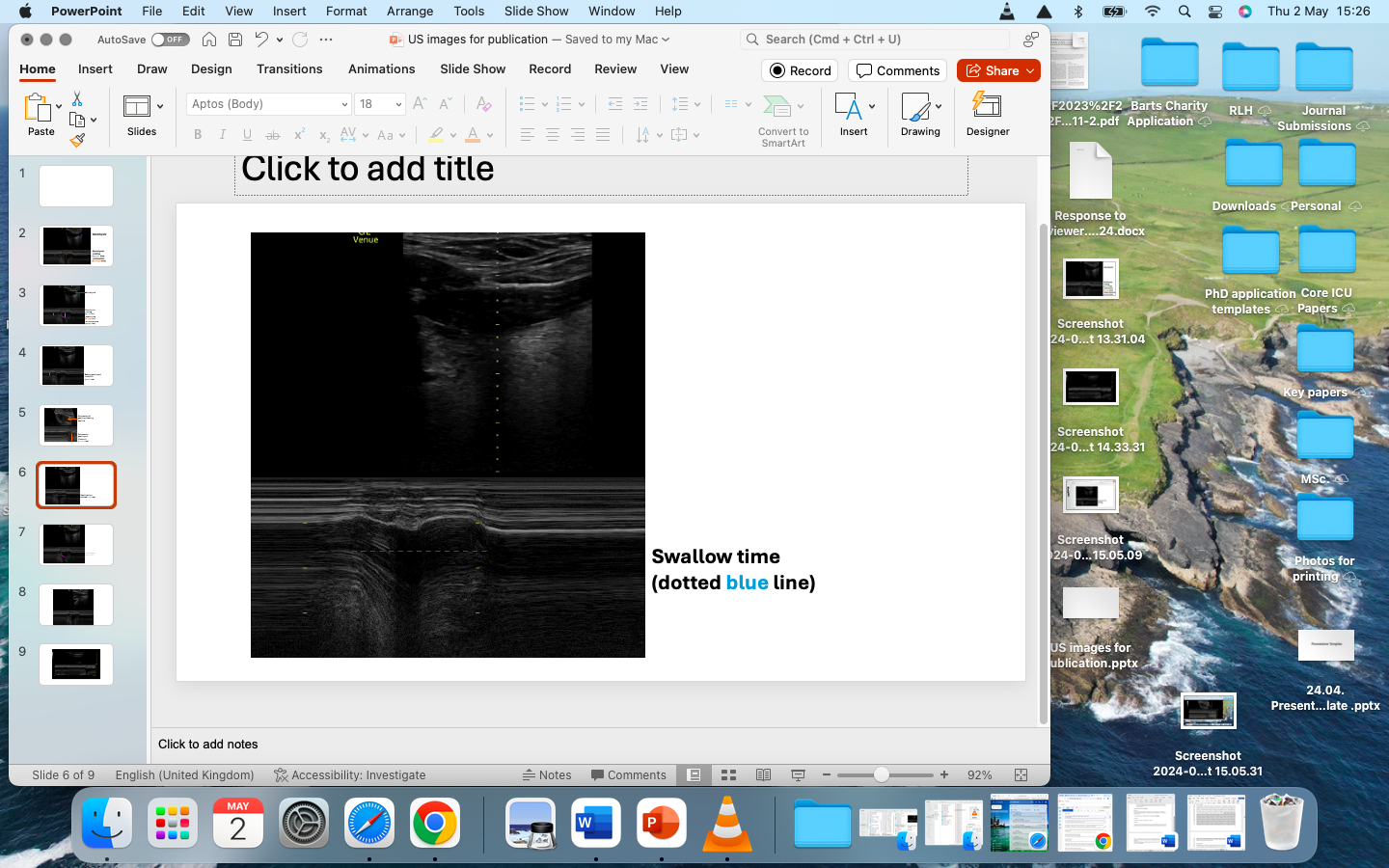


Figure C. Swallow Time

1. **Slope distance and velocity**

Slope distance was measured by calculating the distance between the edge of the angle of the geniohyoid muscle layer at resting position and the deepest point of the geniohyoid muscle layer thickness during maximum contraction. Velocity was calculated automatically by the GE Venue^TM^ ultrasound machine when slope distance was calculated.


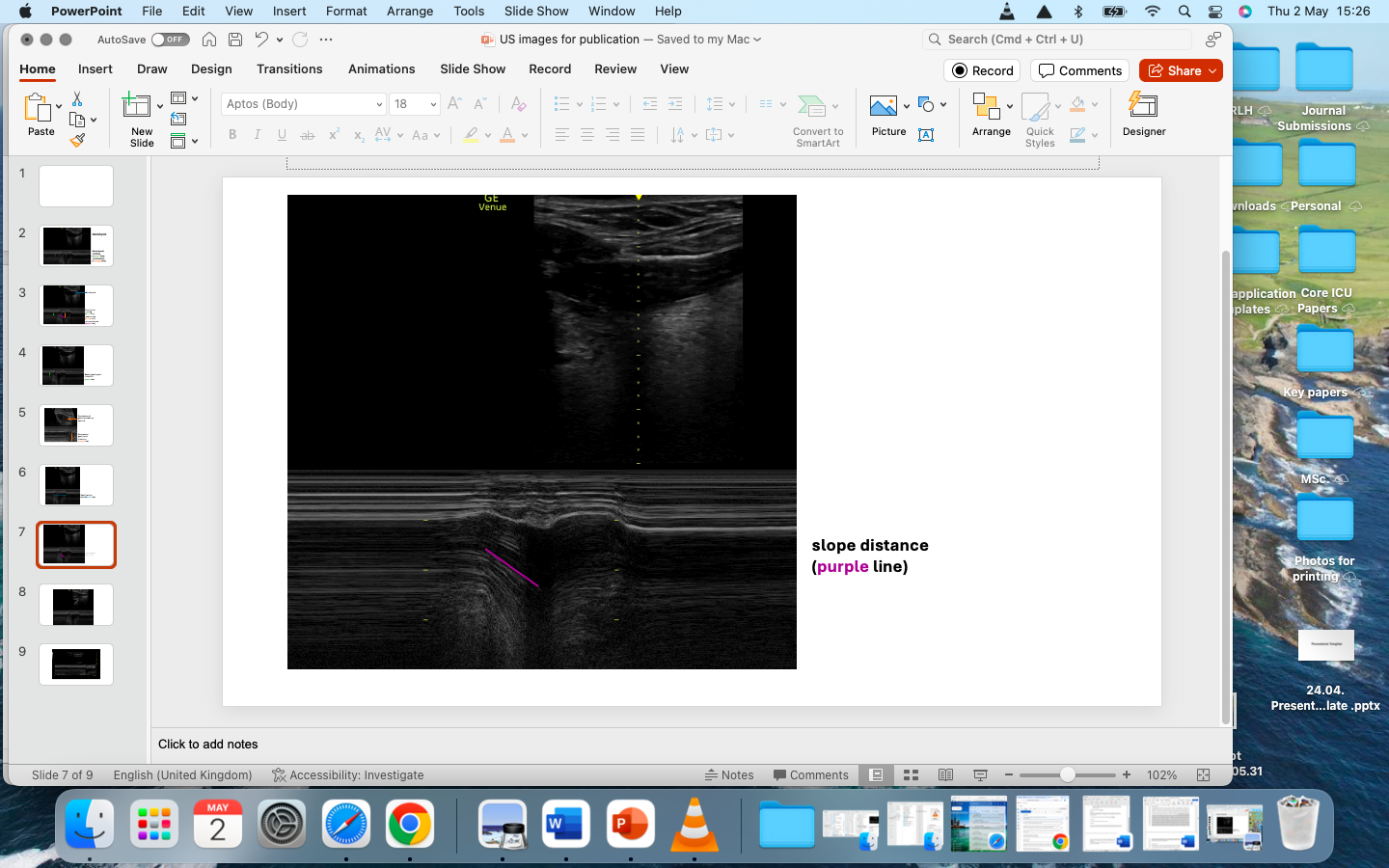


Figure D. Slope Distance
